# MetaDEGalaxy: Galaxy workflow for differential abundance analysis of 16s metagenomic data

**DOI:** 10.12688/f1000research.18866.2

**Published:** 2019-10-18

**Authors:** Mike W.C. Thang, Xin-Yi Chua, Gareth Price, Dominique Gorse, Matt A. Field

**Affiliations:** 1 Institute for Molecular Bioscience, University of Queensland, Brisbane, Queensland, 4000, Australia; 2 Queensland Facility for Advanced Bioinformatics, University of Queensland, Brisbane, Queensland, 4000, Australia; 3 John Curtin School of Medical Research, Australian National University, Canberra, ACT, Australia; 4 Australian Institute for Tropical Health and Medicine, James Cook University, Smithfield, Queensland, 4878, Australia; 5 Centre for Tropical Bioinformatics and Molecular Biology, James Cook University, Smithfield, Queensland, 4878, Australia

**Keywords:** Galaxy, metagenomics, differential abundance, high throughput sequencing, phyloseq

## Abstract

Metagenomic sequencing is an increasingly common tool in environmental and biomedical sciences.  While software for detailing the composition of microbial communities using 16S rRNA marker genes is relatively mature, increasingly researchers are interested in identifying changes exhibited within microbial communities under differing environmental conditions. In order to gain maximum value from metagenomic sequence data we must improve the existing analysis environment by providing accessible and scalable computational workflows able to generate reproducible results.

Here we describe a complete end-to-end open-source metagenomics workflow running within Galaxy for 16S differential abundance analysis. The workflow accepts 454 or Illumina sequence data (either overlapping or non-overlapping paired end reads) and outputs lists of the operational taxonomic unit (OTUs) exhibiting the greatest change under differing conditions. A range of analysis steps and graphing options are available giving users a high-level of control over their data and analyses. Additionally, users are able to input complex sample-specific metadata information which can be incorporated into differential analysis and used for grouping / colouring within graphs.  Detailed tutorials containing sample data and existing workflows are available for three different input types: overlapping and non-overlapping read pairs as well as for pre-generated Biological Observation Matrix (BIOM) files.

Using the Galaxy platform we developed MetaDEGalaxy, a complete metagenomics differential abundance analysis workflow. MetaDEGalaxy is designed for bench scientists working with 16S data who are interested in comparative metagenomics.

MetaDEGalaxy builds on momentum within the wider Galaxy metagenomics community with the hope that more tools will be added as existing methods mature.

## Introduction

It is now recognized that there is a strong link between microbial communities in the human body and human health ^1^. While the importance of such communities is understood, the composition and function of the human micro-biome largely remains a mystery. Uncovering how the composition and function of the micro-biome impacts human health represents a significant area of growth. Another important area of research growth is the study of environmental microbial communities in fields such as agriculture, marine science, and ecology. By identifying the composition of microbial communities, researchers are able to link microbes to specific environments and using comparative metagenomics identify how microbial communities’ changes under altered environmental conditions.

Central to elucidating the link between the metagenomic data and human health or altered environmental conditions is sequencing; however, obtaining useful research outcomes from large volumes of unprocessed sequence data represents a challenge for many bench scientists. The major bottleneck in obtaining value from such data is the huge computational and logistic task required for analysing the large volumes of sequencing data routinely generated in a single sequencing run.

The sequencing of entire microbial communities requires metagenomic analysis tools. These tools rely on the ability to analyse unbroken sequence reads covering the 16S variable regions. Due to limitations of short read sequencing platforms such as IIlumina, the longest fragment of variable regions of a 16S gene that can be sequenced is shorter than the ideal full 600 bp. Illumina paired-end sequencing of 300 bp on forward read and reverse read produces only 550 bp to allow for stitching the forward end and reverse end together. With 550 bp fragment length, the reads can cover both variable region 3 (V3) and variable region 4 (V4). The length of V3 and V4 are 393bp and 440bp respectively.

A major challenge for bench scientists working with metagenomic data is that many popular software programs requires a 64-bit Linux environment, an environment often unavailable and unfamiliar to researchers. Furthermore, even when such an environment is available, the complexity of the rapidly changing metagenomic algorithms means no gold standard methodologies exist. As such, there are currently over 100 metagenomic analysis tools available, making it challenging to select the appropriate software. For example, the popular metagenomic tool QIIME ^2^ consists of more than 150 python scripts, many of which are wrappers to external programs.

An increasingly common alternative for the growing number of non-bioinformaticians working with NGS data is the availability of user-friendly interfaces. These interfaces are typically attached to significant compute resources with pre-installed software packages readily available. Interfaces such as Galaxy ^3^ or the Genomics Virtual Lab ^4^ are examples of powerful platforms that grant non-bioinformaticians access to the latest NGS methodologies. The Galaxy platform enables scientists to use bioinformatics tools in an easy to use graphical user interface (GUI) environment, where tool resource management is handled by the administrators of each Galaxy service. The platform’s functionality power comes from the ability to chain tools into workflows, and share the data and workflows. Further, the flexibility of Galaxy platform allows developers to integrate new tools and workflows into the platform. Galaxy maintains a single tool shed repository of pre-wrapped tools that cover an abundance of next generation sequence analyses.

Despite this, challenges remain in fast moving research areas such as metagenomics with only a handful of complete metagenomic offerings currently available within the popular Galaxy framework. Currently, existing metagenomics options in Galaxy include ASaiM ^5^, FROGS ^6^, GmT ^7^, A-Game ^8^, and ANASTASIA ^9^ with QIIME2 recently becoming available in the Galaxy Toolshed. While there is overlap between their workflows, MetaDEGalaxy differs in its focus on differential abundance by incorporating the capabilities of phyloseq ^10^ and DESeq2 ^11^ for complex differential analysis. DESeq2 contains tests specifically developed to detect differences between groups in abundances for counts data. While DESeq2 is most commonly utilised for differential gene expression in RNASeq, recent studies have shown RNA-Seq algorithms methods perform similarly or better than metagenomic specific algorithms ^12^. MetaDEGalaxy also offers extensive graphing capabilities by wrapping the comprehensive metagenomics R-package phyloseq ^10^. Extensive graphing options are available within MetaDEGalaxy wrapping most functions offered within phyloseq which offer the user a high level of control. Additionally, user supplied metadata files can be input to DESeq2 for model generation and to phyloseq for enhanced graphing capabilities allowing for grouping, clustering, and colouring of all graph types based on metadata information. All software wrapped within the workflow is open-source software, a current limitation of existing workflows such as usearch ^13^ within the popular QIIME package ^2^. Finally, MetaDEGalaxy is designed within the popular Genomic Virtual Lab ^4^ leveraging the functionality of this robust infrastructure.

## Methods

### Input

MetaDEGalaxy accepts either 454 or Illumina paired end sequence FASTQ files that can be overlapping or non-overlapping. Users may alternatively input a pre-computed BIOM file if they do not require BIOM file generation. Additional functionality requires a sample specific tab-delimited metadata file formatted according to QIIME map file standards. This metadata information can be utilised for determining the model to employ within DESeq2 and to generate graphs grouped by various metadata attributes.

### Implementation

In total, there are four workflows in MetaDEGalaxy ( Table 1) which utilise a combination of external software and custom code.

**Table 1.** MetaDEGalaxy Workflows.

Workflow Name	Workflow Description
***1. Quality control and predetermination*** ***of 16S workflow utilisation***	To detect percentage of paired-end reads that overlap each other by 10bp. This workflow randomly selected 1000 reads from each sample to perform the detection. If over 50% of the PE reads overlap each other by at least 10bp, it is recommended to use workflow 2. If less than 50% of PE reads overlap by at least 10bp, it is recommended to use workflow 3.
***2. 16S_DE_for_overlapPE***	For use with datasets that are sequenced using overlapping paired-end reads
***3. 16S_DE_for_nonoverlapPE***	For use with datasets that are sequenced using non-overlapping paired-end reads.
***4. 16S_BIOM***	Handles Biological Observation Matrix (BIOM) file from workflows 2 and 3 to generate 5 plots (e.g. sample correlation network plot, symmetric plot and 3 abundance bar plots.

External software available include Trimmomatic (v0.32.2) ^14^, FastQC (v0.52), PEAR (v0.9.6) ^15^, SAMTools (v1.1.2) ^16^, BWA (0.7.12.1) ^17^, VSEARCH (v1.9.7) ^18^, the BIOM API, DESeq2 (v2.1.8) ^11^ and phyloseq (Galaxy v1.0) ^10^.

### Workflows

Four comprehensive MetaDEGalaxy tutorial are currently available in github which demonstrate how to work with both overlapping and non-overlapping 16S paired end Illumina reads.

**Figure 1.**
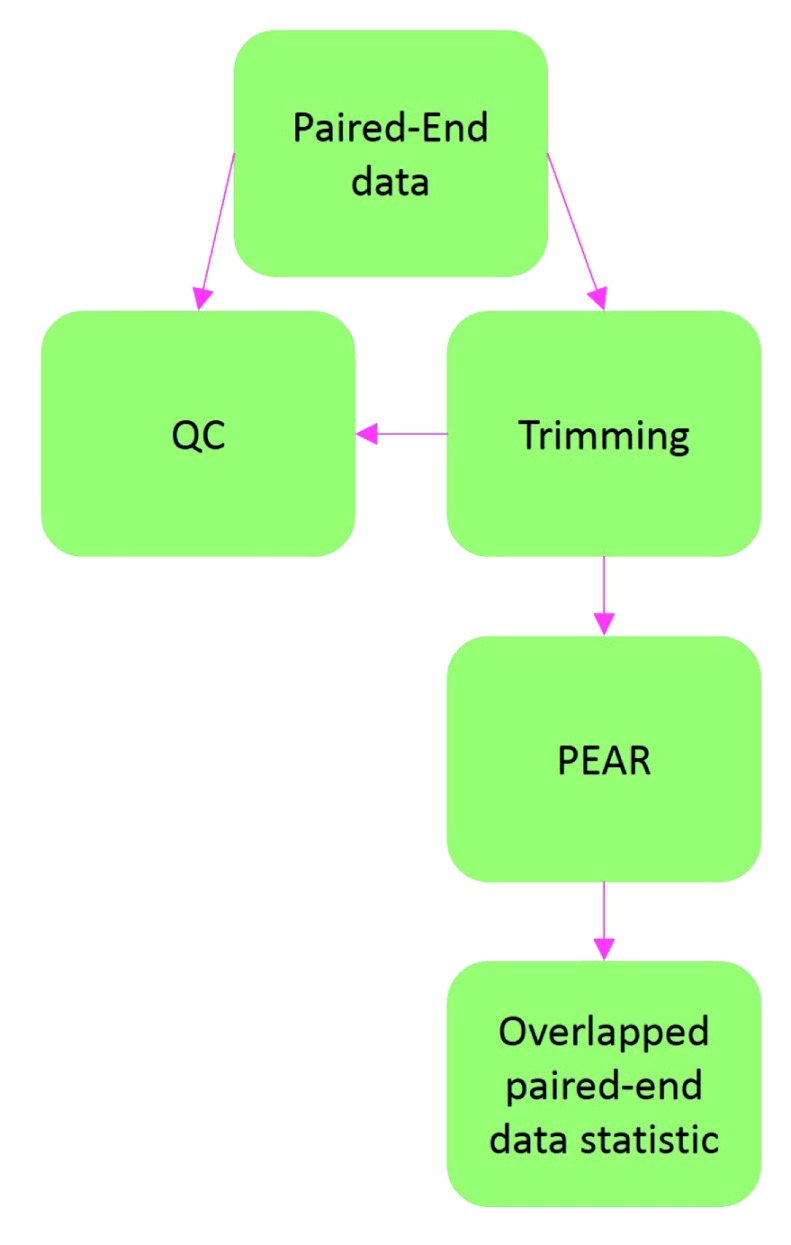
Workflow 1 in MetaDEGalaxy for data QC and detecting PE read overlap.

**Figure 2.**
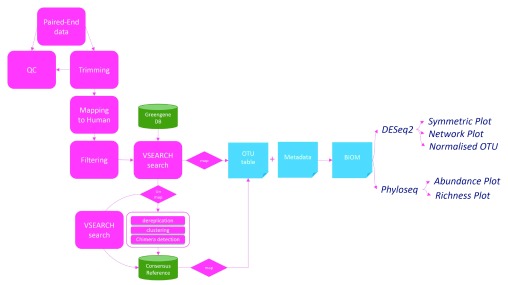
Workflow 2, 3, and 4 for differential abundance detection of operational taxonomic units (OTUs). Both workflow 2 and 3 use all the components in the workflow, the only difference is workflow 2 takes in paired-end reads data as input and workflow 3 take single-end reads data as input. The workflow 4 is the subset of the main workflow which starts with blue boxes and ends with all plots generated.

Tutorial #1 details the workflow for data QC and the detection of paired end overlap in sequencing data and preparing FastQ files for metagenomic analysis ( Figure 1). Tutorial #2 details the entire workflow for overlapping paired end Illumina reads ( Figure 2) using the same data set employed by the Mothur_SOP run with the popular Mothur software (v1.35.1) ^19^. This workflow inputs a group of paired-end MiSeq files and a metadata map file and generates overlapping FASTQ files, an annotated BIOM file, a DESeq2 table of differentially expressed microbes, and a variety of phyloseq graphs. Tutorial #3 details the entire workflow for non-overlapping paired end Illumina reads and is similar to tutorial #2 with the exception of pre-processing steps transforming FASTQ files into a Fasta file where PEAR ^15^ software is not run. Finally, tutorial #4 details a workflow for BIOM file processing and analyses detailing how to utilise the platform for analyses starting from an input BIOM file.

### Operation

The Galaxy environment is available for testing purposes at http://203.101.224.202/galaxy/ and will be available on Galaxy Australia server by the end of 2019 ( https://usegalaxy.org.au/). The minimum system requirements for installing the MetaDEGalaxy are a 64-bit unix environment at 4Gb of memory.

## Results

To demonstrate some of the advanced functionality of MetaDEGalaxy, we follow tutorial #2 using the Mothur_SOP data to first generate a normalised count table and a table of differentially abundant OTUs ( Table 2). The differentially abundant OTU table is formatted in DESeq2 output with additional taxonomic information appended to each row.

**Table 2.** Differentially abundant operational taxonomic units from DESeq2.

OTUID	baseMean	log2FoldChange	lfcSE	stat	pvalue	padj	Kingdom	Phylum	Class	Order	Family	Genus	Species
2916985	17.275	6.959	0.8086	8.606	7.554E-18	4.033E-14	k__Bacteria	p__ Tenericutes	c__Mollicutes	o__RF39	f__	g__	s__
740299	16.78	-6.384	0.7575	-8.429	3.4972E-17	9.336E-14	k__Bacteria	p__Firmicutes	c__Clostridia	o__Clostridiales	f__	g__	s__
274697	16.135	6.822	0.8382	8.139	3.981E-16	7.0848E-13	k__Bacteria	p__Firmicutes	c__Clostridia	o__Clostridiales	f__	g__	s__
641881	25.667	-6.793	0.8645	-7.858	3.9058E-15	5.2132E-12	k__Bacteria	p__Firmicutes	c__Clostridia	o__Clostridiales	f__	g__	s__
697688	17.944	-5.31	0.7502	-7.079	1.4539E-12	1.5524E-09	k__Bacteria	p__Firmicutes	c__Clostridia	o__Clostridiales	f__	g__	s__
778075	11.611	-5.762	0.8519	-6.764	1.3409E-11	1.1932E-08	k__Bacteria	p__Firmicutes	c__Clostridia	o__Clostridiales	f__	g__	s__
134615	6.278	-4.988	0.8125	-6.14	8.277E-10	6.313E-07	k__Bacteria	p__Firmicutes	c__Clostridia	o__Clostridiales	f__	g__	s__
801865	4.222	-5.046	0.8375	-6.026	1.6837E-09	1.1237E-06	k__Bacteria	p__Firmicutes	c__Clostridia	o__Clostridiales	f__	g__	s__
4387364	28.34	-2.546	0.432	-5.894	3.7682E-09	2.2354E-06	k__Bacteria	p__Firmicutes	c__Clostridia	o__Clostridiales	f__	g__	s__
131618	10.229	4.34	0.7439	5.834	5.4269E-09	2.8974E-06	k__Bacteria	p__Firmicutes	c__Clostridia	o__Clostridiales	f__Lachnospiraceae	g__	s__
790360	10.402	3.906	0.692	5.645	1.6498E-08	8.0073E-06	k__Bacteria	p__ Bacteroidetes	c__Bacteroidia	o__Bacteroidales	f__S24-7	g__	s__
1918929	7.056	-5.04	0.8971	-5.618	1.9322E-08	8.2648E-06	k__Bacteria	p__ Proteobacteria	c__ Alphaproteobacteria	o__Rickettsiales	f__mitochondria	g__	s__
352171	7.667	-4.15	0.7397	-5.611	2.0124E-08	8.2648E-06	k__Bacteria	p__Firmicutes	c__Clostridia	o__Clostridiales	f__	g__	s__
790211	17.004	-3.34	0.5968	-5.595	2.2019E-08	8.397E-06	k__Bacteria	p__Firmicutes	c__Clostridia	o__Clostridiales	f__Ruminococcaceae	g__	s__
194043	17.857	3.766	0.688	5.474	4.4026E-08	1.567E-05	k__Bacteria	p__ Bacteroidetes	c__Bacteroidia	o__Bacteroidales	f__S24-7	g__	s__
265712	11.111	-4.232	0.7821	-5.411	6.2728E-08	2.0931E-05	k__Bacteria	p__Firmicutes	c__Clostridia	o__Clostridiales	f__	g__	s__
789537	85.172	2.863	0.5468	5.235	1.648E-07	5.1757E-05	k__Bacteria	p__ Bacteroidetes	c__Bacteroidia	o__Bacteroidales	f__S24-7	g__	s__
799694	3.722	-4.53	0.8702	-5.205	1.9379E-07	5.7482E-05	k__Bacteria	p__Firmicutes	c__Clostridia	o__Clostridiales	f__	g__	s__
761977	3.389	-4.15	0.8112	-5.116	3.1154E-07	8.7542E-05	k__Bacteria	p__Firmicutes	c__Clostridia	o__Clostridiales	f__	g__	s__
674084	17.231	-3.077	0.6053	-5.083	3.7056E-07	9.892E-05	k__Bacteria	p__Firmicutes	c__Clostridia	o__Clostridiales	f__	g__	s__
206817	12.735	3.292	0.6542	5.031	4.8752E-07	0.00011831	k__Bacteria	p__ Bacteroidetes	c__Bacteroidia	o__Bacteroidales	f__S24-7	g__	s__
705799	20.949	-3.175	0.6301	-5.038	4.7021E-07	0.00011831	k__Bacteria	p__Firmicutes	c__Clostridia	o__Clostridiales	f__Ruminococcaceae	g__ Ruminococcus	s__
196733	8.735	3.292	0.6588	4.996	5.8464E-07	0.00013571	k__Bacteria	p__ Bacteroidetes	c__Bacteroidia	o__Bacteroidales	f__S24-7	g__	s__
727165	12.562	3.574	0.7231	4.942	7.7311E-07	0.00017199	k__Bacteria	p__Firmicutes	c__Clostridia	o__Clostridiales	f__Lachnospiraceae	g__	s__
723287	17.06	-3.009	0.6165	-4.881	1.0562E-06	0.00022557	k__Bacteria	p__Firmicutes	c__Clostridia	o__Clostridiales	f__	g__	s__
608864	22.231	-2.95	0.6119	-4.821	1.4262E-06	0.00029286	k__Bacteria	p__Firmicutes	c__Clostridia	o__Clostridiales	f__	g__	s__

**Figure 3.**
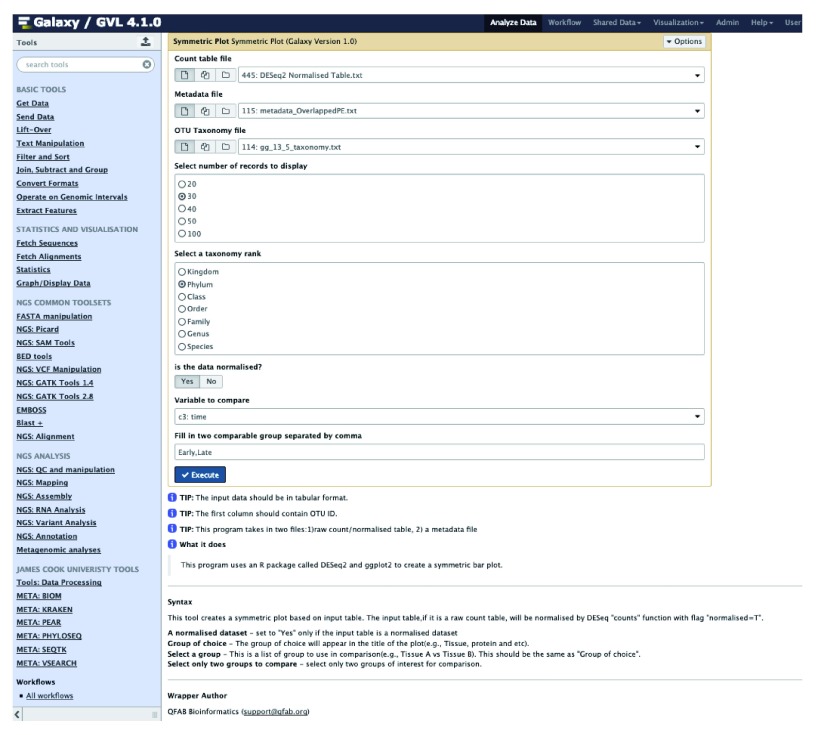
MetaDEGalaxy menu options for generating symmetric plots for differentially abundant operational taxonomic units (OTUs). Users are able to select the taxonomic rank to examine in addition to two values within any user-defined metadata category.

**Figure 4.**
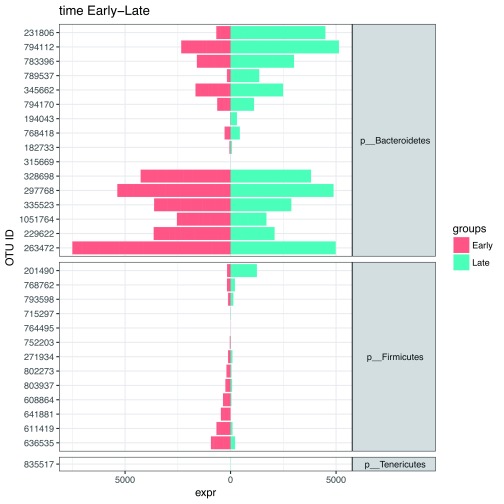
Symmetric plot of the most differentially abundant operational taxonomic units (OTUs) grouped by ‘Time’ with ‘Early’ and ‘Late’ samples compared.

**Figure 5.**
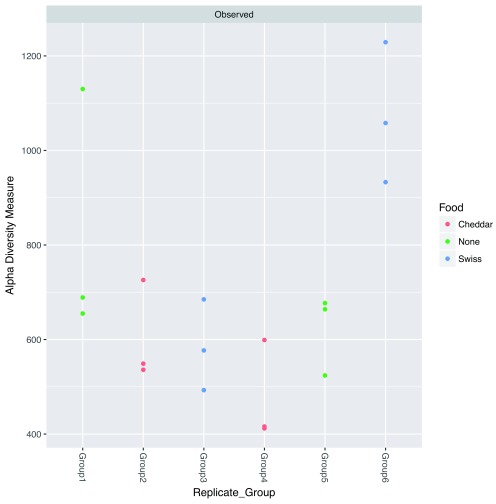
Alpha diversity abundance plots grouped for replicate group and coloured by ‘Food’.

**Figure 6.**
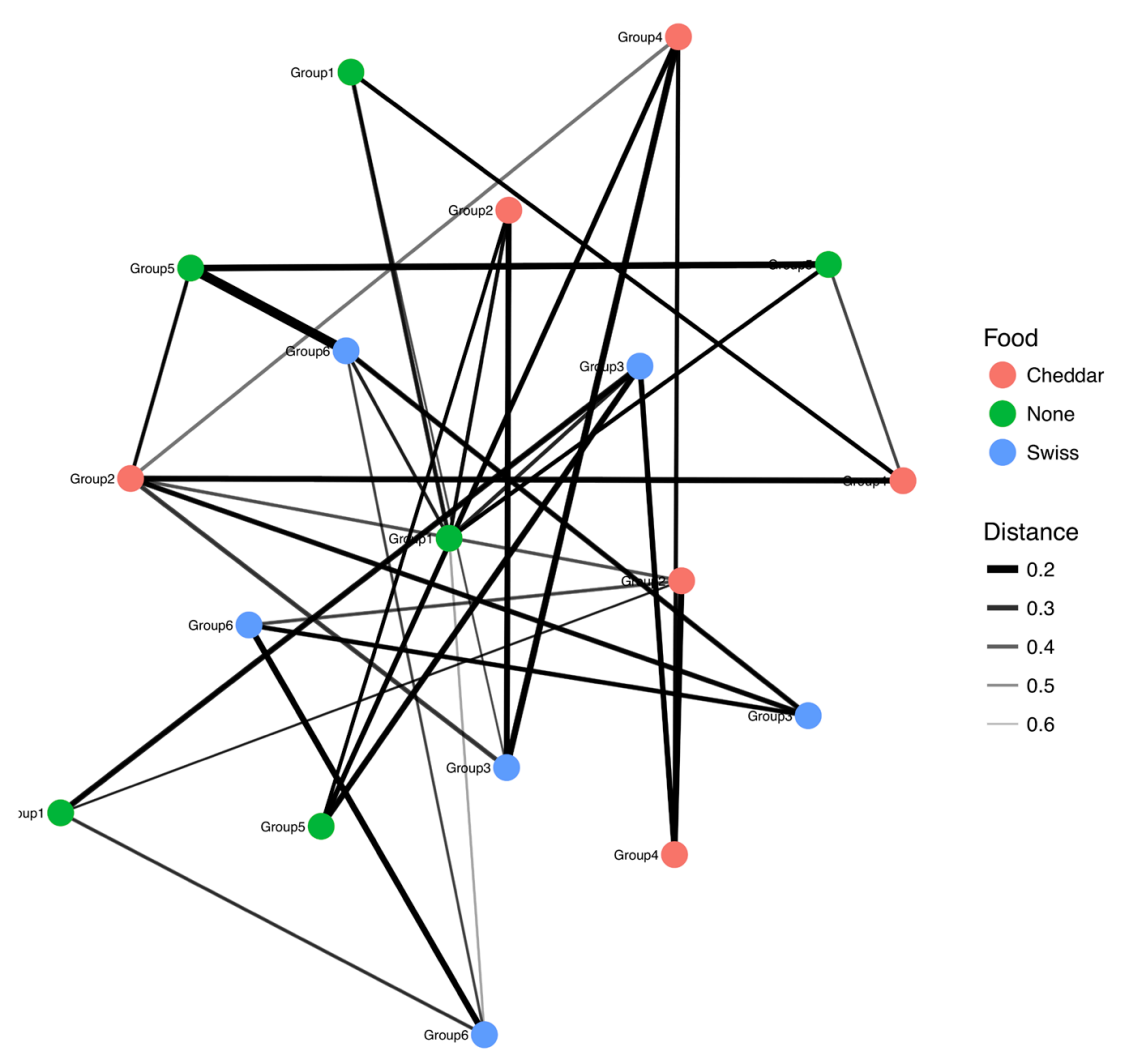
Network plots grouped for replicate group and coloured by ‘Food’.

We use this table of differentially abundant OTUs to next generate a symmetric plot. Users are able to select any taxonomic level as well as any metadata variable for comparison and further to pick two values of this variable for direct comparison ( Figure 3). In this example, we pick Phylum for our taxonomy level and time as our variable of interest and group the graph according to ‘Early’ or ‘Late’. The resulting symmetric plot shows the differences in OTUs for ‘Early’ and ‘Late’ samples across different phylum ( Figure 4). We are also able to generate alpha diversity abundance plots according to various sample attributes grouped here for ‘Replicate Group’ and coloured by ‘Food’ ( Figure 5). As a final example, we generate a network plot where we select ‘Replicate group’ for the correlation and select ‘Food’ as the legend ( Figure 6).

### Software comparison

MetaDEGalaxy is compared to existing software in Table 3. There are comparable web and/or GUI based tools such as QIIME/QIIME2 ^2^, MetaPipe ^20^, MG-RAST ^21^, MOCAT2 ^22^, Calypso ^23^, Explicet ^24^, and Megan ^25^, however none of these tools except QIIME2 are currently available within the popular Galaxy framework. Within Galaxy there are several metagenomics offerings including ASaiM ^5^, GmT ^7^, A-Game ^8^, and ANASTASIA ^9^.

**Table 3.** Popular web or graphical user interface (GUI) based metagenomic analysis pipelines.

Software	Language/Environment	Web?	Input FASTQ?	16S?	Shotgun?	Diff Abun?
QIIME/QIIME2	Python (partial Galaxy)	No	Yes	Yes	Experimental	No
Calypso	Java/Perl/R	Yes	No	Yes	No	Yes
Explicet	C++	No	No	Yes	No	No
Megan	Java	No	No	Yes	No	No
ASaiM	Galaxy	Yes	Yes	Yes	Yes	No
MetaDEGalaxy	Galaxy	Yes	Yes	Yes	No	Yes
Frogs	Galaxy	Yes	Yes	Yes	No	No
MetaPipe	Java/python	Yes	Yes	Yes	Yes	No
MG-RAST	Perl	Yes	Yes	Yes	Yes	No
MOCAT2	Python/Perl	No	Yes	Yes	No	No
ANASTASIA	Galaxy	Yes	Yes	Yes	Yes	No
A-Game	Galaxy	Yes	Yes	No	Yes	No
GmT	Galaxy	Yes	Yes	Yes	No	Partly

While many of the features of the tools overlap, MetaDEGalaxy is the only option within Galaxy combining DESeq2 ^11^ with the full graphing capability of phyloseq ^10^. MetaDEGalaxy is similar in features to GmT ^7^ however the differential abundance options are limited with GmT as it lacks symmetric plots and the ability to construct highly customisable graphs grouped by sample metadata attributes.

Differential abundance tables generated by MetaDEGalaxy and Calypso both use the phyloseq_to_deseq2 function in phyloseq which converts phyloseq formatted BIOM files into a DESeq ready object containing dispersion estimates and an experimental design formula based on a combination of metadata attributes. Mothur differs from these two methods in offering metagenomic specific algorithms including metastat ^26^ and lefse ^27^. Metastats uses a *t*-test with *p*-values derived from an empiric null distribution calculated by sample permutation while lefse applies the non-parametric Kruskal-Wallis and Wilcoxon-Mann–Whitney tests to identify differences in gene abundance between metagenomic groups. Not surprisingly, results from MetaDEGalaxy and Calypso were identical while the results from lefse and metastats were quite different as has been shown by previous studies ^28^.

## Use cases

To demonstrate how to use MetaDEGalaxy we offer four in-depth tutorials describing available workflows. Tutorials 1, 2 and 4 utilise the same input data as the well-documented Mothur_SOP while tutorial 3 utilises custom 300bp paired end, non-overlapping Illumina MiSeq data. In either use case, reads can be accessed and pre-processed via Galaxy Interface with the following steps:

1) click on “Operations on multiple dataset” on the top of the history panel

2) check the box for all paired-end files listed on the history panel

3) click on the "For all selected..." button the top of the history panel

4) click on "Build list of Dataset Pairs" on the drop-down menu

5) Type in a common field of the file name for both forward and reverse paired end data

6) click on the "Auto-pair"

7) Enter a name for the collection of paired datasets and click "Create list"

Apart from the paired-end reads in data collection, users are required to have loaded the metadata table and both 16S reference genome and annotation files. When the paired-end reads from a data collection is imported into a Galaxy history, an important step for the later in the workflow is the renaming of the FASTA sequence header by appending the sample ID to end at the end of each read ID using the reheader tool in Galaxy. This information will be used as the column header for OTU table generated by the workflows.

Workflow 1 ( Figure 1) is designed to detect the status of overlapped paired-end reads data using PEAR. Users should proceed with workflow 2 if the percentage of overlapped paired-end reads data is high. Otherwise, workflow 3 should be used for non-overlapping reads. Both workflow 2 and 3 are fundamentally the same ( Figure 2), however, workflow 3 can take single-end reads data as input when the overlapped paired-end reads are not overlapping.

Workflow 4 is designed to take a precomputed BIOM file as input. BIOM file format is designed to store OTU counts, metadata, and OTU annotation into one file. When users input a BIOM file, workflow 4 can be used to add metadata to an existing BIOM file and create abundance bar plot, network plot and symmetric plots using phyloseq R package.

More detailed tutorial documentation is available in the github repository.

## Conclusion

MetaDEGalaxy is a complete end-to-end Galaxy workflow for 16S differential abundance analysis. Harnessing the power of open source algorithms such as vsearch, phyloseq, and DESeq2, MetaDEGalaxy offers users high-level of control over their data and analysis options. Focusing on discovering the most differentially abundant OTUs between samples, MetaDEGalaxy allows users to assess the impact of different environmental condition on overall microbial community composition.

## Data availability

### Source data

Data used for the tutorials are available from Zenodo:

Zenodo: Mothur MiSeq SOP Galaxy Tutorial Data. https://doi.org/10.5281/zenodo.800651 ^29^

Data are available under the terms of the Creative Commons Attribution 4.0 International license (CC-BY 4.0).

## Software availability

Software available from: http://203.101.224.202/galaxy/

Source code available from: https://github.com/QFAB-Bioinformatics/jcu.microgvl.ansible.playbook

Archived source code at time of publication: https://doi.org/10.5281/zenodo.2658835 ^30^

Licence: GNU General Public License v3.0 for all script/wrappers
