# Supplementary material for: MetaDEGalaxy: Galaxy workflow for differential abundance analysis of 16s metagenomic data
Source: F1000Res. 2019 Oct 18;8:726. Originally published 2019 May 23. [Version 2] doi: 10.12688/f1000research.18866.2 (PMC6807170; doi:10.12688/f1000research.18866.2)
Supplement: Peer review history

# Peer review: Reviewer response for version 2

**DOI:** 10.5256/f1000research.23147.r55374

**Published:** 2019-10-21

**Authors:** Saskia Hiltemann

**Affiliations:** 1 Department of Bioinformatics, Erasmus University Medical Center, Rotterdam, The Netherlands

Thanks to the authors for their revision. All my previous concerns have now been addressed, and I am happy to approve this manuscript.

 Minor suggestions:

1. Be consistent about capitalisation, e.g. fasta, FASTA, FastQ, FASTQ both appear.
2. github -> GitHub, unix -> UNIX, vsearch -> VSEARCH

I confirm that I have read this submission and believe that I have an appropriate level of expertise to confirm that it is of an acceptable scientific standard.

# Peer review: Reviewer response for version 1

**DOI:** 10.5256/f1000research.20677.r55128

**Published:** 2019-10-15

**Authors:** Leo Lahti

**Affiliations:** 1 Department of Future Technologies, University of Turku, Turku, Finland

This submission introduces MetaDEGalaxy, which is a workflow intended for 16S differential abundance analysis in the open source Galaxy platform. The workflow incorporates various currently popular open source algorithms, the proposed workflow support the application of

 such methods by Galaxy users. In particular, the workflow supports differential OTU abundance testing for common measurement platforms (454 and Illumina). Step-by-step tutorials are provided to support the use.

 The overall work is sound and clearly written. Appropriate references are provided, and the work is based on commonly used methodologies and open source resources. Data and software are openly available with a unique DOI and permanent archiving through Zenodo.

 The work does not contribute to methods criticism, validation, or benchmarking.  This work is a technical contribution that provide new software plugin for the broader Galaxy platform. This is relevant for the limited community of researchers who use Galaxy for 16S microbiome analysis. The contribution is a contribution to scientific software, rather than scientific discussion. This, in my understanding, fits the F1000Research scope.

**Minor:**

- Why the software has GPL3 license that is more restrictive than e.g. MIT which is often recommended for research software? See DOI: 10.1371/journal.pcbi.1002598 ^1^
- Instead of QIIME, it could be more appropriate to cite QIIME2?

I confirm that I have read this submission and believe that I have an appropriate level of expertise to confirm that it is of an acceptable scientific standard.

## References

[1] : A quick guide to software licensing for the scientist-programmer. *PLoS Comput Biol*.2012;8(7) : 10.1371/journal.pcbi.1002598 e1002598 10.1371/journal.pcbi.1002598 22844236PMC3406002

# Peer review: Reviewer response for version 1

**DOI:** 10.5256/f1000research.20677.r50121

**Published:** 2019-07-24

**Authors:** Saskia Hiltemann

**Affiliations:** 1 Department of Bioinformatics, Erasmus University Medical Center, Rotterdam, The Netherlands

The authors describe MetaDEGalaxy, a set of Galaxy tools and workflows for differential abundance analysis of 16S metagenomics data. They have enabled DESeq -a tool designed primarily for RNASeq data- to be used on metagenomics datasets. They provide Galaxy workflows and training materials and a Galaxy server for testing. Furthermore, they have integrated a number of Galaxy tools for visualisation using phyloseq, which is a valuable addition to the existing ecosystem of Galaxy metagenomics tools.

**Remarks:**

1. In the abstract: "Metagenomic sequencing [..] analysis workflows remain immature compared to other fields"
  This is a strong claim and requires more justification or be made less broad. Metagenomics (and especially 16S metagenomics) tool suites such as QIIME and Mothur have quite well-established pipelines. And end-to-end online analysis portals such as MG-RAST ( https://www.mg-rast.org/), MGnify ( https://www.ebi.ac.uk/metagenomics/pipelines/2.0), MOCAT2 ( https://mocat.embl.de/), META-pipe ^1^ and others have also been around for some time, and are also user-friendly GUI options.
  In general, the discussion of existing methods could be expanded. Please describe in more detail how MetaDEGalaxy fits in this ecosystem.
2. In the introduction: "Currently, there is one end-to-end existing metagenomics workflow offering, ASaiM":
  a. Referring to ASaiM as a workflow may be confusing. In Galaxy, the term workflow has a very specific meaning, and ASaiM is a collection of tools, workflows and tutorials with a common topic, and it includes multiple Galaxy workflows within it. Perhaps refer to these solutions as Galaxy environments or similar, and reserve the word workflows for Galaxy workflows?
  b. ASaiM is also by no means the only metagenomics workflow available in Galaxy, for example:
  - GmT : Mothur SOP 16S end-to-end pipelines have been made available as Galaxy workflows previously. ^2^
  - FROGS: Metagenomics pipelines in Galaxy has been previously described. ^3^
  - Other Galaxy environments and workflows such as A-Game, ^4^ ANASTASIA, ^5^ or this functional annotation workflow, ^6^ and several others have also been previously described. While these examples have a different focus than MetaDEGalaxy (i.e. functional metagenomics rather than 16S), the authors make the very broad claim that ASaiM is the only other "existing metagenomics workflow on offering", which is inaccurate.
  Please consider expanding the discussion of existing work and Table 3 to include some or all of the above.
3. In the results section, please discuss how the differential abundance results obtained with the MetaDEGalaxy pipelines compare to the results described in the Mothur SOP ( https://www.mothur.org/wiki/MiSeq_SOP, e.g. under subsection "population level analysis" of section "OTU-based analysis") Do you determine the same OTUs to be statistically significantly different between the two groups? Explain any differences in results, as well as the added value of your approach over the statistical methods used in the SOP.
4. The Phyloseq wrappers the authors have created do not appear to be available from the Galaxy toolshed currently While I appreciate that the authors have developed Ansible playbooks for the installation of the wrappers, such a custom approach is not recommended practice, and adding the tools to the tool shed will greatly increase their accessibility.
  One option for this would be to submit the wrappers to the IUC tool repository on github ( https://github.com/galaxyproject/tools-iuc) where they will be reviewed and automatically uploaded to the toolshed upon acceptance.

**Minor Remarks:**

1. Training materials for the use of the MetaDEGalaxy workflows are available in the form of PDF files. I would strongly urge the authors to consider contributing these materials to the Galaxy Training Network ( https://training.galaxyproject.org) so that they are more readily available for use by the community. I think that MetaDEGalaxy tutorial would be happily accepted there, and the GTN community can provide support to transform the tutorials into the right format.
2. Since the 4 workflows offered in this manuscript are designed to be run in succession (e.g. workflow 1,2,4 or 1,3,4), have the authors considered creating some full end-to-end workflows, using Galaxy's concept of sub-workflows?
3. A set of Qiime2 Galaxy tool wrappers have recently been made available in the toolshed ( https://toolshed.g2.bx.psu.edu/repository?repository_id=7af460fa907bf4a3), could you update he text in the "software comparison" section & table to reflect this?

**Compliments:**

 I really like the visualisation tools you added, and I would love to add a symmetric plot to the existing 16S Galaxy tutorial on the Galaxy Training Networks site if you put the tools on the tool shed!

I confirm that I have read this submission and believe that I have an appropriate level of expertise to confirm that it is of an acceptable scientific standard, however I have significant reservations, as outlined above.

## References

[1] : META-pipe cloud setup and execution. *F1000Res*.2017;6: 10.12688/f1000research.13204.3 10.12688/f1000research.13204.3 31069047PMC6480938

[2] : Galaxy mothur Toolset (GmT): a user-friendly application for 16S rRNA gene sequencing analysis using mothur. *GigaScience*.2019;8(2) : 10.1093/gigascience/giy166 10.1093/gigascience/giy166 PMC637740030597007

[3] : FROGS: Find, Rapidly, OTUs with Galaxy Solution. *Bioinformatics*.2018;34(8) : 10.1093/bioinformatics/btx791 1287-1294 10.1093/bioinformatics/btx791 29228191

[4] : A-GAME: improving the assembly of pooled functional metagenomics sequence data. *BMC Genomics*.2018;19(1) : 10.1186/s12864-017-4369-z 10.1186/s12864-017-4369-z 29329522PMC5767027

[5] : ANASTASIA: An Automated Metagenomic Analysis Pipeline for Novel Enzyme Discovery Exploiting Next Generation Sequencing Data. *Front Genet*.2019;10: 10.3389/fgene.2019.00469 469 10.3389/fgene.2019.00469 31178894PMC6543708

[6] : A Galaxy Workflow for the Functional Annotation of Metagenomic Samples.2012;7297: 10.1007/978-3-642-30448-4_31 247-253 10.1007/978-3-642-30448-4_31

# Response

**Published:** 2019-10-16

**Authors:** Matt Field

**Affiliations:** James Cook University, Australia

Thank you for taking the time to review MetaDEGalaxy. I have submitted a modified manuscript to address the points you raised.

1) Software background / comparison (Major Remark 1 and 2 and minor remark 3)

The most significant change to the manuscript is the dramatic expansion of the software discussion and comparison sections to include more web based and Galaxy based metagenomics offerings. Additions include MG-RAST, MetaPipe, MOCAT2, FROGS, GmT, A-Game, and ANASTASIA to name a few.

I added a broader discussion about where MetaDEGalaxy fits in relative to the ever expanding metagenomic software environment.

2) Expansion of differential abundance comparison

I expanded the manuscript to include more details on tools with differential abundance options including calypso and mothur methods metastats and lefse. I performed a small side-by-side comparison however MetaDEGalaxy had results identical to calypso (which also uses phyloseq_to_deseq) and very different to both mothur techniques so I simply state this fact in the manuscript and cite previous work that has shown this already (Jonsson V, et al. Statistical evaluation of methods for identification of differentially abundant genes in comparative metagenomics. BMC Genomics. 2016).

3) Availability of wrapper scripts and tutorials (Major remark 4 and minor remark 1)

Thank you for the suggestions regarding better ways to make the software more widely available, I wasn't aware of these resources.  The tutorials will indeed be made widely available to the training network once the installation is completed within Galaxy Australia which should be done by the end of 2019.  Currently, the code is installed on a demo server however it will be given a permanent home within Galaxy Australia very shortly. We will also make the wrapper scripts available as per your suggestion to the IUC tool repository on github.

Also, for testing please note the temporary IP address for the demo server changed to http://203.101.224.202/galaxy/ which is now reflected in the new manuscript.
